# scGeno: a Hidden Markov Model approach to denoise chromosome-scale genotypes from single-cell data

**DOI:** 10.1093/bioadv/vbag094

**Published:** 2026-04-03

**Authors:** Rosaria Tornisiello, Helene Kretzmer

**Affiliations:** Hasso Plattner Institute for Digital Engineering, Digital Engineering Faculty, University of Potsdam, Potsdam, Germany; Max Planck Institute for Molecular Genetics, Berlin, Germany; Department of Mathematics and Computer Science, Freie Universität Berlin, Berlin, Germany; Hasso Plattner Institute for Digital Engineering, Digital Engineering Faculty, University of Potsdam, Potsdam, Germany; Max Planck Institute for Molecular Genetics, Berlin, Germany

## Abstract

**Motivation:**

Single-cell analysis of monoallelic expression and genomic imprinting requires accurate genotype determination at the cellular level. However, genotype inference from single-cell RNA sequencing data is challenging due to technical noise, allelic dropout, and sparse gene expression patterns, particularly in genetically heterogeneous populations.

**Results:**

Here, we present scGeno, a categorical Hidden Markov Model that infers chromosome-level genotype states in organisms with mixed genotypes by modeling sequential gene expression ratios from single-cell RNA sequencing data. Our method leverages the sequential continuity of the genotype states along chromosomes to overcome single-cell data limitations and generates chromosome-resolved, comprehensive genotype maps for individual samples. Our probabilistic framework accounts for technical noise while maintaining high accuracy in genotype assignment. Validation on experimental data demonstrates robust performance in determining clear genotypic states, thereby enabling systematic investigation of allele-specific expression patterns at single-cell resolution.

**Availability and implementation:**

scGeno is an open-source Python package under an MIT license. Source code, documentation, and installation instructions can be downloaded from GitHub (https://github.com/RosariaTornisiello/Genotype_HMM.git).

## 1 Introduction

**Figure 1:**
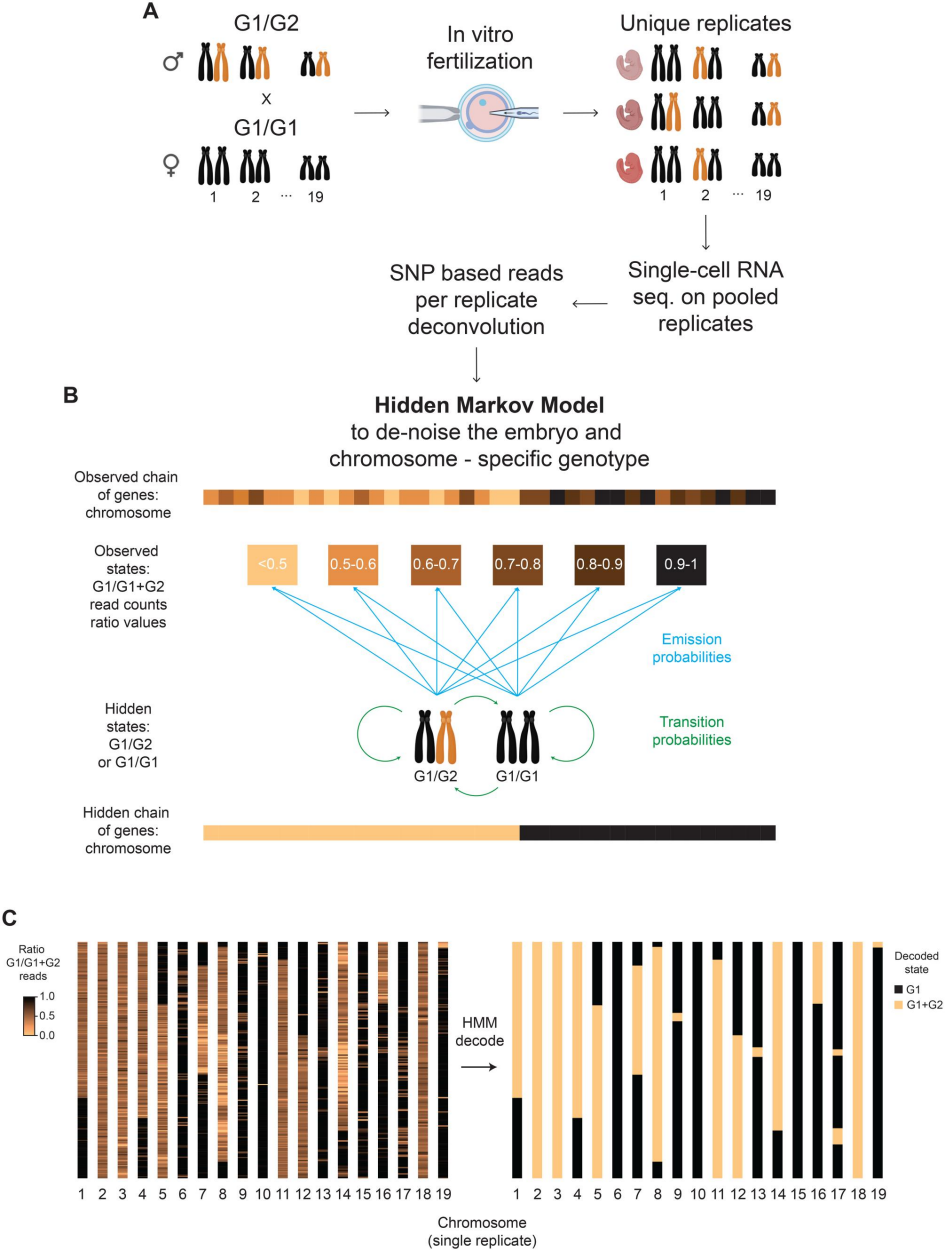
scGeno HMM model overview and application. A. Schema of an example experimental setup to generate replicate-powered F1 hybrid organisms, apply scRNA-seq, deconvolute single replicates, and assign sequencing reads to the specific genotypes used. B. scGeno HMM model implementation and parameters. C. Example of single replicate chromosome maps before (left) and after (right) segmentation using scGeno.

Genomic imprinting is an epigenetic phenomenon by which the paternally or the maternally inherited allele of specific genes is preferentially expressed. This process is observed in many species, including mammals, and its dysregulation has been connected to cancer (Whitworth *et al*. 2024) and other diseases (Lee *et al*. 1999). Moreover, it has been shown that imprinted genes can also be expressed in a tissue-specific manner (Hu *et al*. 1998). A promising strategy to characterize tissue-specific imprinting patterns is to analyze single-cell transcriptomic data obtained from organisms with mixed genotypic backgrounds, as found in F1 hybrids with genomically distinct maternal and paternal alleles, ideally sequenced in multiple replicates. Such data allow allele-specific expression to be traced to the parental origin (G1 or G2) and were already published by us and others (Yin *et al*. 2019, Grosswendt *et al*. 2020, Anderson *et al*. 2021; Simon *et al*. 2024, Wang *et al*. 2024, Petrova *et al*. 2025). In our design, we generated scRNA-seq data from pooled sibling organisms derived from G1/G1 mothers and mixed genotype (G1/G2 F1) fathers, enabling in silico identification of individual replicates based on their stochastically inherited G2 alleles (Fig. 1A). In principle, this experimental framework allows for examining imprinting; however, three key challenges are inherent to the experimental setup. First, the Mendelian segregation patterns in the crossing design led to the random assortment of G1/G2 heterozygous and G1/G1 homozygous chromosomal states within individual replicates. This stochastic distribution of chromosomes results in heterozygous allele combinations that are not uniformly present across all replicates, thus limiting the systematic investigation of monoallelic gene expression in a replicate-specific, random manner. Second, chromosomal crossover events occurring during meiosis in the heterozygous F1 males lead to recombination between G1 and G2 homologous chromosomes, resulting in sperm that inherit paternal chromosomes exhibiting segmental mosaicism with alternating G1 and G2 genotypic regions. Third, the inherent sparsity of scRNA-seq data makes the precise definition of chromosomal genotype maps challenging. Several bioinformatics tools have leveraged Hidden Markov Models (HMMs) to computationally reconstruct genome-wide haplotypes from sequencing data. For instance, TIGER (Rowan *et al*. 2015) infers genotypes from low-coverage Whole Genome Sequencing (WGS) in F2 mapping populations, while GBRS (Choi *et al*. 2025) reconstructs founder haplotype mosaics directly from bulk RNA-seq in multiparent mouse populations. These approaches demonstrate the power of HMMs for haplotype phasing but are limited to bulk data with relatively high coverage. While such tools have great power, none exist to generate chromosome block-resolved genotype maps for individual scRNA-seq samples. To address these limitations, we developed scGeno, a probabilistic framework based on a categorical HMM that models individual chromosomes as sequential chains of discrete genetic events from single-cell expression ratios, with each gene representing a single observational unit. Our algorithm infers the inherent sequential correlation structure along chromosomes from allele-specific gene expression data, including breaks in the genotype likely caused by crossover events. Combined with replicate and cell-type assignment of cells, this allows for the analysis of comprehensive allele-specific expression maps of individual replicates. Taken together, scGeno effectively denoises noisy genotype information and generates high-confidence predictions of chromosomal genotype segmentation patterns.

## 2 Implementation

### 2.1 Input data

The user should generate separate G1- and G2-specific feature-barcode matrices for each sample, using G1 or G2-specific, known SNPs to assign individual reads to one of the two genomic backgrounds. For each replicate, scGeno calculates the allelic bias estimates (expression ratios) r at the single-cell level and for each gene as follows:


$$r=\frac{G1}{G1+G2}$$

Then, the mean ratio across all cells of a replicate is obtained as the replicate-level allelic bias estimate per gene, which is skewed to 0.5 in a G1/G2 and 1 in a G1/G1 background region.

### 2.2 Parameters

To implement the scGeno HMM model (Fig. 1B), the observed states are defined by categorizing the allelic bias estimates (r) into six discrete observed states based on predefined intervals: r < 0.5, 0.5–0.6, 0.6–0.7, 0.7–0.8, 0.8–0.9, and 0.9–1.0. All estimates below 0.5 are grouped into a single observed state based on the biological rationale that such values predominantly arise from heterozygous G1/G2 chromosomal segments, where balanced allelic expression or G2-biased expression would yield ratios in this range. In our model, the hidden states represent the true underlying genomic architecture, consisting of either homozygous G1/G1 regions, where only G1 alleles are present, or heterozygous G1/G2 segments containing one allele from each parental strain. All HMM parameters (initial, transition, and emission probabilities) are initialized before training and iteratively updated during the learning process. Initial probabilities are set to 0.5 for both hidden states, reflecting the equal likelihood that the first gene on each chromosome could originate from either of the two genotypic configurations. Transition probabilities are configured to reflect the biological expectation of rare recombination events along chromosomes: the probability of transitioning between G1/G1 and G1/G2 states was set to 0.002. In contrast, the probability of remaining in the same hidden state was set to 0.998. This parameterization is informed by the biological knowledge that mouse chromosomes contain approximately 1,000 protein-coding genes on average and experience roughly two crossing-over events per chromosome during meiosis (Paigen *et al*. 2008), yielding an expected transition rate of 2/1,000 = 0.002 per gene. This concept could also be generalized to other mammals since they share similar patterns and frequency of meiotic recombination events (Dumont 2017).

### 2.3 Training

To optimize parameter estimation, the model is trained on randomly selected chromosomes pooled from all replicates. Users can specify the number of chromosomes per replicate used for training. While parameter values show limited variation with respect to training set size, they stabilize after the inclusion of ∼100 sequences (Supplementary Figure 1A-C, available at *Bioinformatics Advances* online). The trained HMM is then employed to decode the chromosome chains and assign all detected genes to a specific genotype state (Fig. 1C).

## 3 Results

### 3.1 Benchmark

To benchmark scGeno’s performance, we analyzed publicly available scRNA-seq data of wild-type mouse embryo samples (Grosswendt *et al*. 2020). Upon visual inspection of the decoded sequences, the trained model shows optimal segmentation capabilities, producing well-defined genotype boundaries along chromosomes, suggesting that the model has successfully learned the underlying genotype structure and the relationship between the hidden and the observed chains (Fig. 1C). To validate model performance without ground truth annotations, we employed posterior predictive checks (PPCs), generating synthetic sequences from the fitted model for comparison with real observations. The simulated data show mean values (real = 2.47, simulated = 2.63), standard deviations (real = 2.77, simulated = 2.19), and transition rates (real = 0.41, simulated = 0.42) similar to those of the real data, indicating that scGeno captures the core patterns of the real chromosome segmentations. However, they show a significantly different distribution of emitted states (Kolmogorov-Smirnov (K-S) test: statistic = 0.032815, p-value < 0.05) and median to the real data (Mann-Whitney U test: statistic = 4247516464.00, p-value < 0.05, Supplementary Figure 1D-E available at *Bioinformatics Advances* online), most likely reflecting unmodeled scRNA-seq noise and biological variability while preserving haplotype switching statistics. To further validate the model, we compared its performance across varying gene-per-chromosome percentages (40, 60, or 80% of the genes) for both training and testing, simulating reduced heterozygous SNP coverage. Specifically, we trained the reduced scGeno models using the same random gene subset across all replicates and then compared the resulting models to the original one (“full”). All the resulting models’ parameters showed high Pearson correlation coefficients with each other and with the full model (Supplementary Figure 3A, available at *Bioinformatics Advances* online), with low mean squared error (MSE) and mean absolute difference (MAD) as compared to the distribution of the full model parameters (40%: MAD = 0.026, MSE = 0.002, 60%: MAD = 0.030, MSE = 0.002, 80%: MAD = 0.033, MSE = 0.003). These results suggest that varying the number of instances of the training sequences did not affect the learned probabilities. However, when decoding new sequences using the same down-sampling technique, these models underperformed relative to the full model. For one representative replicate, we decoded all chromosome sequences across the three reduced scGeno model variants and computed the percentage of genes per chromosome with consistent observed state assignments (Supplementary Figure 3B, available at *Bioinformatics Advances* online). Across model versions, these percentages remained consistently low (mean across chromosomes: 40% model: 43.2%; 60% model: 47.7%; 80% model: 47%). These results indicate that scGeno’s decoding performance improves when the genetic divergence between G1 and G2 is highest, as reflected by a larger number of heterozygous SNPs. In other words, scGeno is better able to distinguish and accurately assign alleles when many informative heterozygous sites are available between the two genomes.

### 3.2 Application

As a proof of concept, we compared wild-type data with knock-out mouse embryo samples (Grosswendt *et al*. 2020). Specifically, we selected knock-out data of the DNA-methyltransferase 1 (Dnmt1), an enzyme responsible for maintaining methylation imprints during mouse preimplantation development (Hirasawa *et al*. 2008). After segmenting the genome of each embryo using scGeno, based on the genotype background, we analyzed the expression of known imprinted genes, using only replicate embryos for which the imprinted genes of interest were localized within a mixed-genotype segment. Known maternally and paternally imprinted genes exhibited higher expression from the respective parental allele in wild-type samples. In contrast, Dnmt1 knockout samples displayed a more balanced expression of both parental alleles (Supplementary Figure 2A, available at *Bioinformatics Advances* online) (KS test paternal genes p-value 4.177e-06, KS test maternal genes p-value 2.20e-10). Additionally, known ubiquitously expressed, non-imprinted genes show only minor fold changes between the two different genotypes (KS test control genes p-value: 9.801e-1, Supplementary Figure 2B, available at *Bioinformatics Advances* online). Moreover, our analysis confirmed some known tissue-specific imprinted genes. For example, the imprinted genes Commd1, Slc22a2, and Copg2 have been reported to be biallelically expressed in the placenta (Extra-embryonic ectoderm, or XEctoderm). Similar expression patterns were found for Nap1l5 and Peg13, for which no imprint was reported in the extraembryonic tissues, and Magel2, which is known to be imprinted at Embryonic day 17.5 but not earlier (Tunster *et al*. 2013), display a fold-change in expression close to 0 (Supplementary Figure 2C, available at *Bioinformatics Advances* online). Finally, genes previously reported to be imprinted in all tissues, like Cdkn1c, H19, Ppp1r9a, Igf2, and others, exhibit extreme fold changes, confirming scGeno’s high sensitivity (Supplementary Figure 2C, available at *Bioinformatics Advances* online).

## 4 Discussion

Parent-of-origin-specific gene expression and its tissue-specific regulation can be explored systematically using single-cell RNA sequencing data from F1 hybrids with distinct parental alleles. However, accurately inferring genotypes from such data remains challenging due to sparse expression, allelic dropout, and chromosomal recombination. Although a few computational pipelines exist to investigate allele-specific expression from WGS (Rowan *et al*. 2015) and bulk RNA-seq data (Choi *et al*. 2025), no method operating on scRNA-seq data has been developed to reconstruct chromosome-level genotype maps as a framework for evaluating tissue-specific mono-allelic expression. To address this gap, we developed scGeno, a categorical hidden Markov model that infers chromosome-level genotype states by modeling sequential allelic expression ratios. scGeno accurately reconstructs genotype segmentation and detects crossover events in noisy single-cell data. Validation of wild-type and Dnmt1-knockout embryos confirmed biologically consistent imprinting patterns, implying robust genome segmentations. While scGeno demonstrates robust performance, a few limitations inherent to the input data and modeling approach should be considered. The scRNA-seq intrinsic low allelic coverage results in high allelic dropout rates, which can overestimate apparent genotype switches and reduce segmentation accuracy, particularly in lowly expressed genomic regions. Additionally, scGeno’s HMM-based sequential modeling of the chromosome maps relies exclusively on the detected genes expressed in the specific sample analyzed; therefore, it cannot directly infer the genotypes of non-coding regions, gene deserts, centromeres, and telomeres. Lastly, biological confounders should be considered, *e.g.*, SNP density heterogeneity across the genome poses a challenge for sequential HMM modeling, especially for state transitions, and several mechanisms of mono-allelic expression can produce imbalanced allelic ratios. However, the chromosomal context modeling of scGeno mitigates these local confounders. Future improvements could incorporate allele-specific dropout correction and variable emission probabilities modeling to handle allelic imbalance. Beyond its current applications, scGeno establishes a framework for integrating genotype inference into allele-specific, sparse single-cell analyses. This paves the way for studies of mosaicism, chimerism, and genotype-phenotype relationships in complex contexts.

## Supplementary Material

vbag094_Supplementary_Data

## Data Availability

The data underlying this article have been previously published and are available in the Gene Expression Omnibus (GEO) under GSE137337.
