# Supplementary material for: scGeno: a Hidden Markov Model approach to denoise chromosome-scale genotypes from single-cell data
Source: Bioinform Adv. 2026 Apr 3;6(1):vbag094. doi: 10.1093/bioadv/vbag094 (PMC13075984; doi:10.1093/bioadv/vbag094)
Supplement: vbag094_Supplementary_Data [file vbag094_supplementary_data.pdf]

scGeno: a Hidden Markov Model approach to denoise chromosome-scale genotypes from single-cell data

Authors: Rosaria Tornisiello[1,2,3], Helene Kretzmer[1,2]

Affiliations:

[1]Hasso Plattner Institute for Digital Engineering, Digital Engineering Faculty, University of Potsdam, Potsdam, Germany

[2]Max Planck Institute for Molecular Genetics, Berlin, Germany

[3]Department of Mathematics and Computer Science, Freie Universität Berlin, Berlin, Germany

Supplementary information

Experimental approach

In brief, we generated single-cell RNA sequencing (scRNA-seq) data from pooled sibling embryos derived from mixed genotype (B6/CAST F1) fathers, enabling in silico identification of individual replicates based on their stochastically inherited CAST alleles and sex determination through X- and Y-linked gene expression profiles. This experimental approach has been used by us (Grosswendt *et al.* 2020) to demultiplex cells by embryo replicate from a pooled single-cell RNA sequencing experiment.

Input data

Cell-to-embryo assignment was performed using autosomal CAST Single Nucleotide Polymorphism (SNP) content as detailed in (Grosswendt *et al.* 2020), yielding separate B6- and CAST-specific feature-barcode matrices for each experimental sample. Following quality control filtering to retain genes and cells present across the two allele-specific matrices and an aggregated one, data were partitioned by individual replicate embryo. For each embryonic replicate, we computed the allelic expression ratio B6/(B6+CAST) at the single-cell level for each gene, then calculated the mean ratio across all cells within the respective replicate to obtain replicate-level allelic bias estimates.

Supplementary Figures

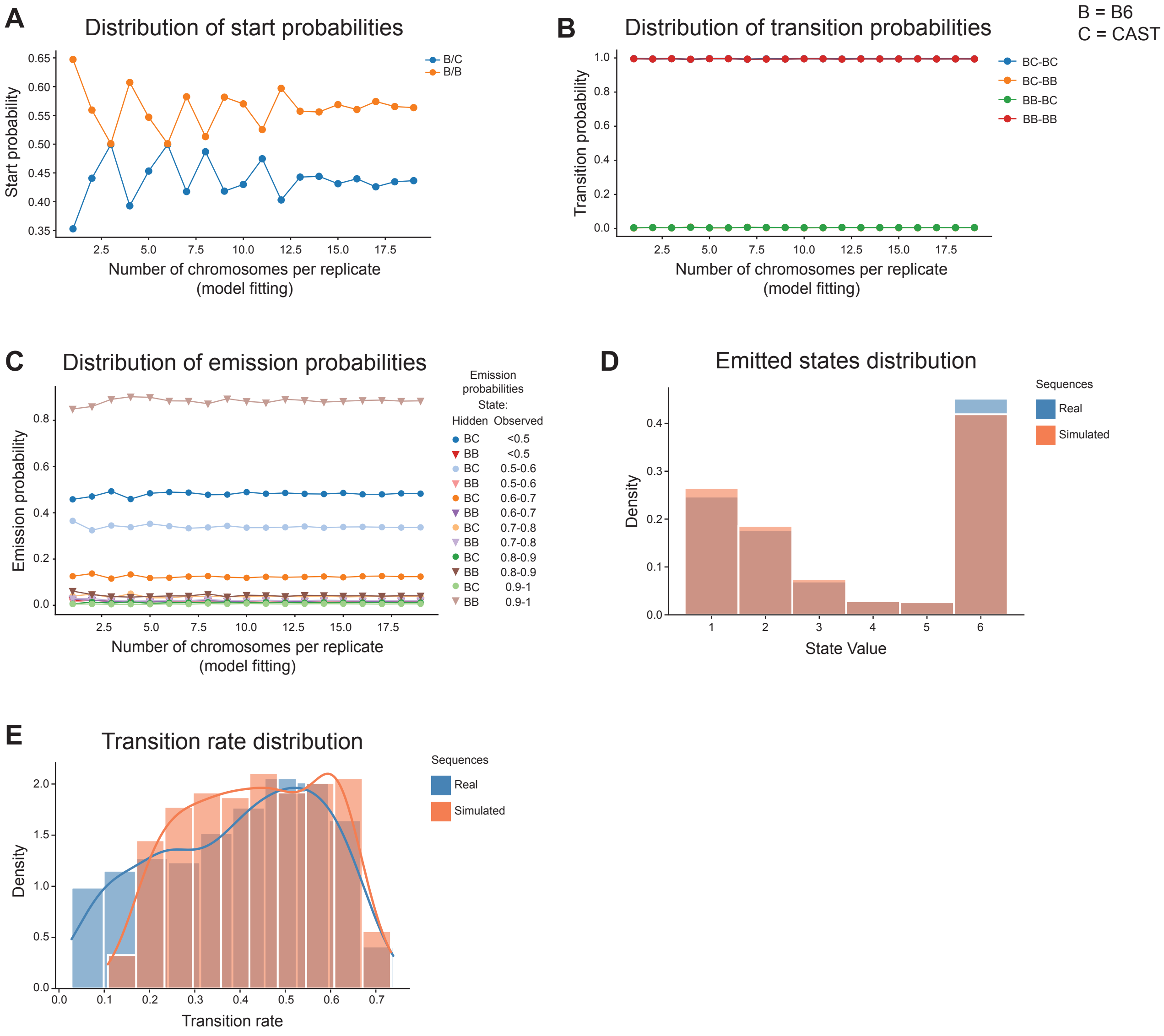

Supplementary Figure 1 – scGeno HMM parameters estimation and Post Predictive Checks **A.** Distribution of start probabilities, **B.** distribution of transition probabilities and **C.** distribution of emission probabilities, over the size of the training set, described as the number of random chromosomes per replicate (in this case, replicates = 10). **D.** Emitted states distribution and **E.** Transition states distribution of real data examples (blue) and simulated data generated using our trained scGeno HMM. Here, "B" = B6 and "C" = CAST, the two genotypes used in this dataset.

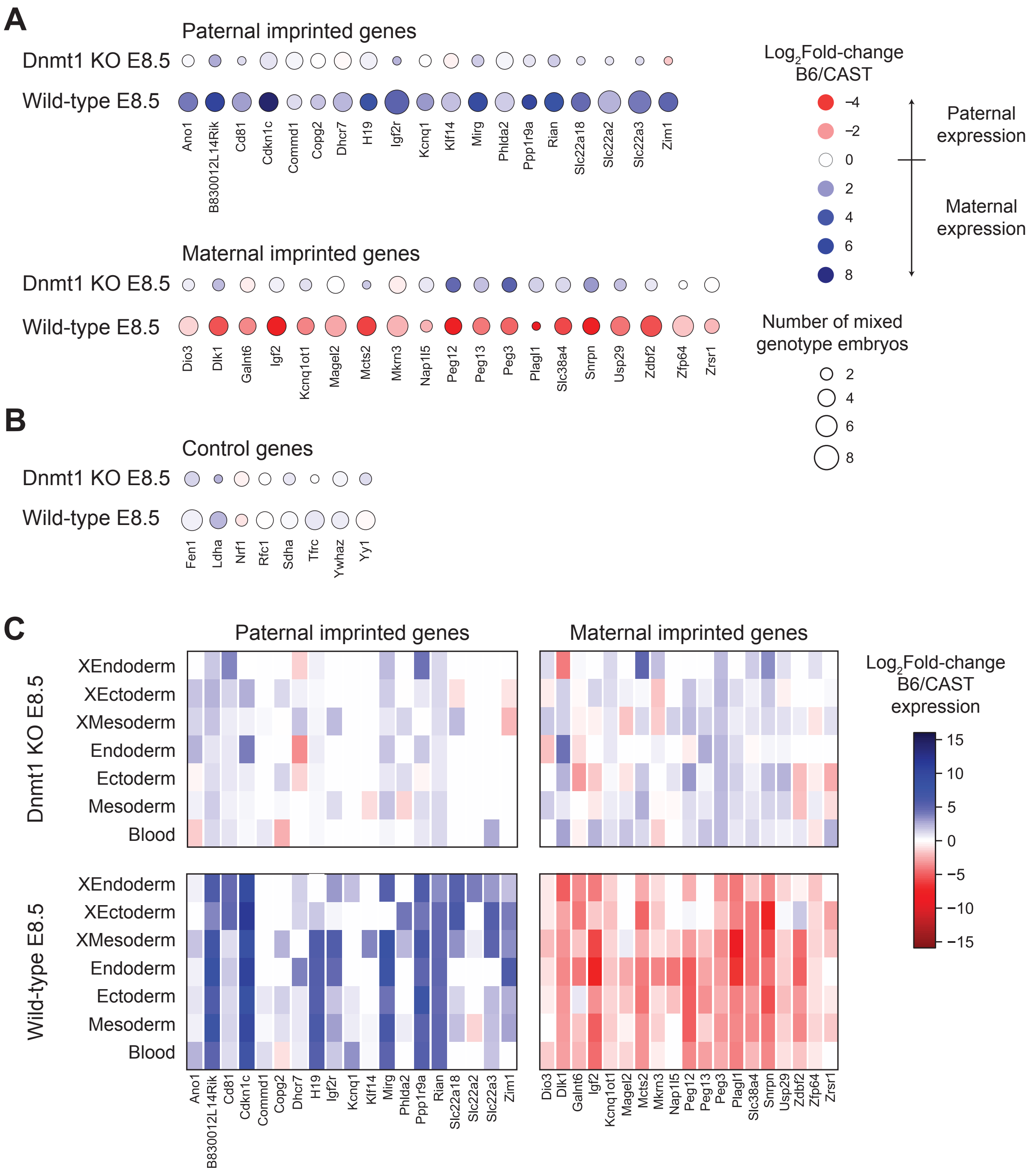

Supplementary Figure 2 – Example application of scGeno and imprinted gene expression analysis using publicly available scRNA-seq data of wild-type and Dnmt1 knock-out mouse embryos. **A.** Examples of known paternal and maternal imprinted genes: the color code indicates the Log_2 (B6/B6 + CAST) expression in Dnmt1 knock-out mouse embryos and wild-type mouse embryos at Embryonic stage 8.5, while the size of the dots shows the number of replicate embryos in which each gene is found in a mixed genotype segment. **B.** Same plot as a. but for control, non-imprinted genes. **C.** Heatmaps showing the same data as a and b, but in a tissue-specific manner (rows). Here, the “X” prepended to the tissue name indicates “Extra-embryonic”.

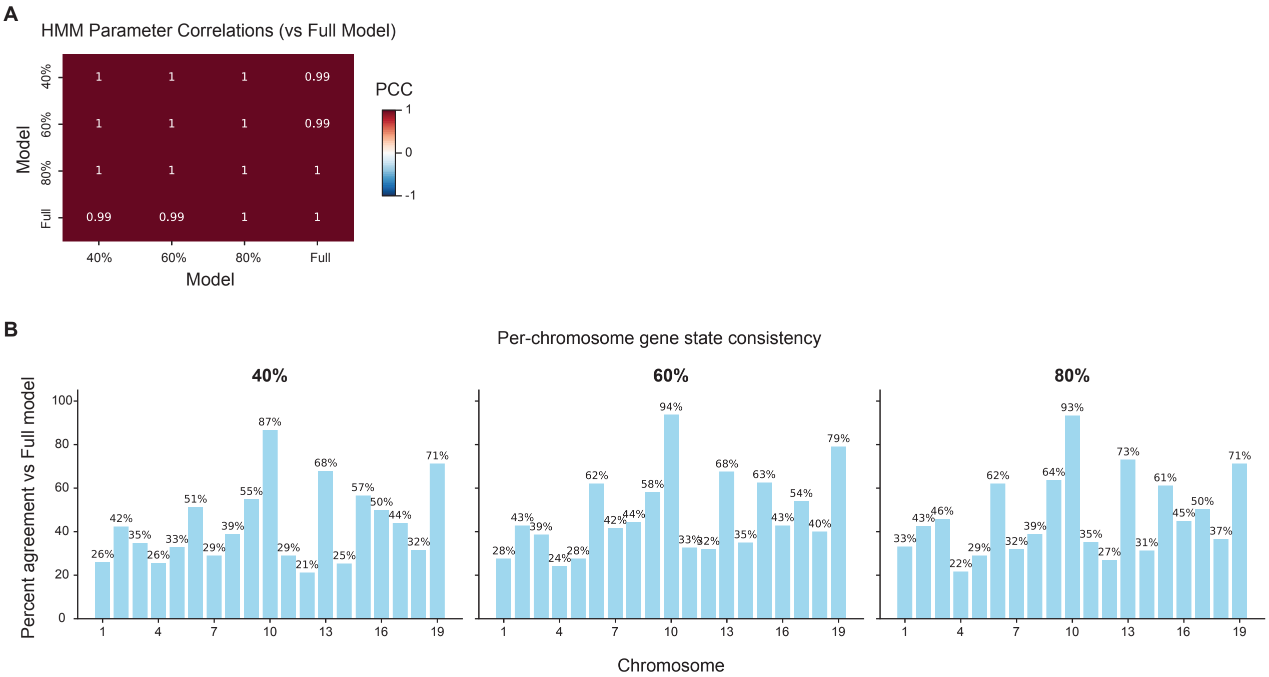

Supplementary Figure 3 – Model evaluation at different SNP densities. **A.** Pearson Correlation Coefficient (PCC) of scGeno's model parameters at different gene densities as an approximation for 40%, 60% and 80% SNP densities. The full model, including all genes available, is included. **B.** Percentage of genes per chromosome showing consistent state assignment across the three reduced scGeno models (40, 60, 80% of all expressed genes), in the decoded sequence of an example replicate.
